# Antitoxin at Colchester

**Published:** 1902-04-12

**Authors:** 


### Antitoxin at Colchester.

An unfortunate difference has occurred between the medical officer of health of Colchester and certain members of the Town Council. From a speech made by Alderman Laver (consulting surgeon to the Essex and Colchester Hospital) at a meeting of the Colchester Town Council last week we gather that the question hinged chiefly upon the use of antitoxin in the treatment of the hospital patients. According to the report in the *Essex County Standard*, Alderman Laver, commenting on Dr. Brown's annual report, and referring to the acknowledged importance of giving antitoxin as early as possible, asked Dr. Brown "whether he thought that the administration of antitoxin as he gave it in the majority of his cases (from two to ten days after their admission) was giving it at the earliest possible stage of the disease, or whether it was not an example of a perfunctory administration of a remedy by an unbeliever in its value, which vitiated all the conclusions which he drew from his figures." Now we are not going to follow this quarrel. It is quite clear to us that the medical officer of a public institution is absolutely and solely responsible for the medical treatment of the patients within it, and that any attempt on the part of any such body as a Town Council or a Health Committee to dictate to him the line of treatment which he is to adopt must end in disaster. On the other hand, a public body, on becoming cognisant that a line of treatment is being adopted in a hospital under its charge which varies materially from that which is generally adopted by the profession, is perfectly within its rights in taking steps to bring about a change of *personnel*. The complications which have occurred at Colchester are among the almost necessary consequences of what we venture to call the bad system of throwing upon the medical officer of health, who should be a sanitary specialist, the purely medical work of attending the patients in the fever hospital; for it must frequently happen that a very good sanitary officer may lose the confidence of the people in regard to the medical treatment which he adopts, so different are the two kinds of work which it is sought to combine in one office, the holder of which must indeed be an Admirable Crichton if he is able to perform all its duties with success.

At Colchester, the feeling in favour of a more complete and early recourse to antitoxin has no doubt been greatly emphasised by the remarkable report lately issued by the Colchester Town Council, and signed by Dr. Louis Cobbett, of Cambridge, and Dr. G. S. Graham-Smith, respecting the measures taken to check the outbreak of diphtheria in 1901. The number of cases notified amounted to 286, and of these 200 cases were treated in the Infectious Hospital. From January 1st to July 16th (previous to which date antitoxin was not systematically used in the Infectious Hospital) 89 cases were admitted, of whom 21 died, or 23·59 per cent. From July 16th to December 28th 113 patients were admitted, of whom 8 died, or 7·8 per cent. Outside the hospital the number of cases in the first period was 37 with 4 deaths, or a mortality of 10·8 per cent.; while during the second period there were 46 cases, of whom 6 died, or 13·13 per cent. Thus it would appear that at a time when the outside fatality was rising, that within the hospital was greatly lowered on the systematic employment of antitoxin. We gather, moreover, that the case was really much stronger than these figures indicate, for of the 89 patients admitted during the first period, 15 were known to have had antitoxin administered before their admission, and of these none died; while during the second period two patients at the hospital were treated without antitoxin by their private medical attendants, and of them one died. When the correction for these cases has been made it appears that of the antitoxin cases 5·6 per cent. died, while of those treated by other methods 28·9 per cent. succumbed. Nor was the remarkable fall in the fatality of the hospital cases which occurred after the introduction of antitoxin a gradual diminution, for we are told that a carefully compiled chart of the weekly admissions and deaths shows that the change was abrupt, the introduction of the new treatment being followed by 60 cases without a single death, while at the same time the outside mortality, by which the severity of type may be estimated, kept at a higher level than before. With these figures before them we do not wonder at the people of Colchester being converted to the antitoxin treatment.

